# Characterization of Pathogenicity-Associated V2 Protein of Tobacco Curly Shoot Virus

**DOI:** 10.3390/ijms22020923

**Published:** 2021-01-18

**Authors:** Mingjun Li, Changchang Li, Kairong Jiang, Ke Li, Junlei Zhang, Miao Sun, Gentu Wu, Ling Qing

**Affiliations:** Chongqing Key Laboratory of Plant Disease Biology, College of Plant Protection, Southwest University, Chongqing 400716, China; LCC130916005@163.com (C.L.); kairongj11@163.com (K.J.); lihai8730@163.com (K.L.); jleiZHANGg@163.com (J.Z.); sunmiao4458@163.com (M.S.); wugtu@163.com (G.W.)

**Keywords:** tobacco curly shoot virus, V2, RNA silencing suppressor, pathogenicity

## Abstract

V2 proteins encoded by some whitefly-transmitted geminiviruses were reported to be functionally important proteins. However, the functions of the V2 protein of tobacco curly shoot virus (TbCSV), a monopartite begomovirus that causes leaf curl disease on tomato and tobacco in China, remains to be characterized. In our report, an *Agrobacterium* infiltration-mediated transient expression assay indicated that TbCSV V2 can suppress local and systemic RNA silencing and the deletion analyses demonstrated that the amino acid region 1–92 of V2, including the five predicted α-helices, are required for local RNA silencing suppression. Site-directed substitutions showed that the conserved basic and ring-structured amino acids in TbCSV V2 are critical for its suppressor activity. Potato virus X-mediated heteroexpression of TbCSV V2 in *Nicotiana benthamiana* induced hypersensitive response-like (HR-like) cell death and systemic necrosis in a manner independent of V2′s suppressor activity. Furthermore, TbCSV infectious clone mutant with untranslated V2 protein (TbCSV_∆V2_) could not induce visual symptoms, and coinfection with betasatellite (TbCSB) could obviously elevate the viral accumulation and symptom development. Interestingly, symptom recovery occurred at 15 days postinoculation (dpi) and onward in TbCSV_∆V2_/TbCSB-inoculated plants. The presented work contributes to understanding the RNA silencing suppression activity of TbCSV V2 and extends our knowledge of the multifunctional role of begomovirus-encoded V2 proteins during viral infections.

## 1. Introduction

RNA silencing is a highly conserved mechanism that plays important roles in endogenous gene regulation and primary antiviral response in eukaryotes [1,2]. To counter plant virus infection, Dicer-like (DCL) proteins of host recognize the double-stranded (ds) RNA molecular structures such as viral replication intermediates or structured viral RNAs, which are subsequently cleaved into small interfering RNAs (siRNAs) with 21–24 nucleotide (nt) in length and are referred to as virus-derived siRNAs (vsiRNAs) [3,4]. Then, one strand of the double-stranded siRNA is integrated into the argonaute (AGO)-containing RNA-induced silencing complex (RISC) for degradation of cognate RNA molecules, such as the vsiRNA-deriving virus or the endogenous RNA with sequence complementation [5,6,7]. During the arms race between viruses and hosts, at least one viral suppressor of RNA silencing (VSR) was evolved by most of the known viruses [8,9,10]. These VSRs can target diverse steps of RNA silencing through different mechanisms to suppress antiviral defense of plant hosts [11,12,13,14,15].

An increasing number of reports have revealed that special sequence motifs or amino acids of some VSRs play crucial roles in active RNA silencing suppression. The suppressors p1 of sweet potato mild mottle virus (SPMMV) and p0 of potato leafroll virus (PLRV) contain GW/WG motifs that were reported to be crucial for AGO1 targeting and ultimately RNA silencing suppression [16,17]. P21 encoded by beet yellows virus (BYV) can bind siRNA in vitro, and the arginine at the position of 90 (R90), R130 and polar residue T149 were presumed to be important for RNA binding based on the crystal structure analysis [18]. The R2 and R86 located in the putative RNA binding inner surface of grapevine leaf roll associated virus-2 (GLRaV-2) p24 are necessary for the suppressor activity [19]. The asparagine residue in the FRNK box of Hc-Pro encoded by papaya ringspot virus is critical for its function to bind small RNA and suppress PTGS [20]. 

Plant-infecting geminiviruses with circular single-stranded DNA (ssDNA) genome are important causal agents of various viral diseases worldwide, which lead to extensive agriculture production losses, especially in tropical and subtropical regions [21]. Geminiviruses were classified into nine genera; among them, *Begomovirus* is the largest genus, with more than 400 members [22]. Partial begomoviruses only comprise one circular ssDNA as genome, named monopartite begomoviruses, and a circular ssDNA satellite molecule was selectively associated with them for infection [21,23]. Six proteins (V1 and V2 from the viral strand, and C1, C2, C3 and C4 from the complementary strand) are encoded by circular genomic DNA and at least one protein named βC1 can be encoded by the satellite DNA [24,25]. Increasing evidence unraveling the pathogenicity mechanisms recruited by viral proteins of begomoviruses with (such as tomato yellow leaf curl China virus and the betasatellite complex, TYLCCNV/TYLCCNB) or without satellite (such as tomato yellow leaf curly virus, TYLCV) for successful infections has been reported in the last several decades [26,27,28,29,30]. For tobacco curly shoot virus (TbCSV), some isolates in the field are associated with a betasatellite ((tobacco curly shoot betasatellite, TbCSB), and coinfection with TbCSB can intensify symptom severity in tobacco hosts. However, the knowledge on the function of viral proteins encoded by TbCSV is limited [31,32,33]. 

To our knowledge, independent studies have identified that V2, C1, C2, C4/AC4 and βC1 encoded by different geminiviruses can suppress PTGS and/or TGS [31,32,34,35,36,37,38,39,40]. Besides, previous reports have shown that V2 proteins encoded by begomoviruses with or without betasatellite have diverse functions for infection. TYLCV and cotton leaf curl Kokhran virus (CLCuKoV) encoded V2 proteins were reported to be involved in viral movement, and loss-of-function of CLCuKoV V2 led to significant decrease of viral accumulation and symptomless phenotype on inoculated plants [41,42,43]. As reported previously, the most common characteristics of V2 are hypersensitive response (HR) induction and RNA silencing suppression. Potato virus X (PVX) mediated heterologous expression of V2 encoded by papaya leaf curl virus (PaLCuV), tomato leaf curl Java virus-A (ToLCJV-A[ID]) or CLCuKoV can induce a hypersensitive response [44,45]. V2 proteins encoded by TYLCV and some other begomoviruses were reported to function as suppressors of PTGS and/or TGS [44,46,47,48,49,50,51]. Nevertheless, the RNA-silencing suppressor activity of V2 was virus-specific. V2 encoded by watermelon chlorotic stunt virus (WmCSV), a bipartite begomovirus, was unable to suppress exogenous GFP-induced RNA silencing [48]. A previous report showed that the C2 and C4 proteins of TbCSV are VSRs; however, it is still unknown if TbCSV V2 could also be a functionally diverse VSR and function in viral pathogenicity [32]. In addition, if the HR-like response induced by PVX-mediated expression of V2 associated with V2′s suppressor activity remains to be investigated.

In this study, we showed that TbCSV V2 can suppress positive-sense GFP RNA-induced local and systemic RNA silencing, and the conserved amino acid region 1–92, which contains five predicted α-helices, is required for VSR activity of V2. Site-directed mutagenesis showed that basic and ring-structured amino acids located in the conserved region (aa 1–92) of V2 are crucial for RNA silencing suppression. Notably, R17 and R43, which may be involved in RNA binding and V2′s self-interaction, respectively, are crucial for suppressor activity of V2. We also found that PVX-mediated expression of TbCSV V2 can induce HR and systemic necrosis in *N. benthamiana*, and this function is independent of the VSR activity of V2. The TbCSV mutant with loss-of-function of V2 (TbCSV_∆V2_) failed to induce any visible symptom throughout the infection stage, and the accumulation of mutant virus was decreased significantly. Coinfection of TbCSV_∆V2_ with betasatellite (TbCSB) could obviously elevate the viral accumulation and induce symptom at the early infection stage, whereas the symptom recovery on the systemically infected leaves occurred over time.

## 2. Results

### 2.1. TbCSV V2 Inhibited Both Local and Systemic RNA Silencing Triggered by Positive-Sense GFP RNA

**Figure 1:**
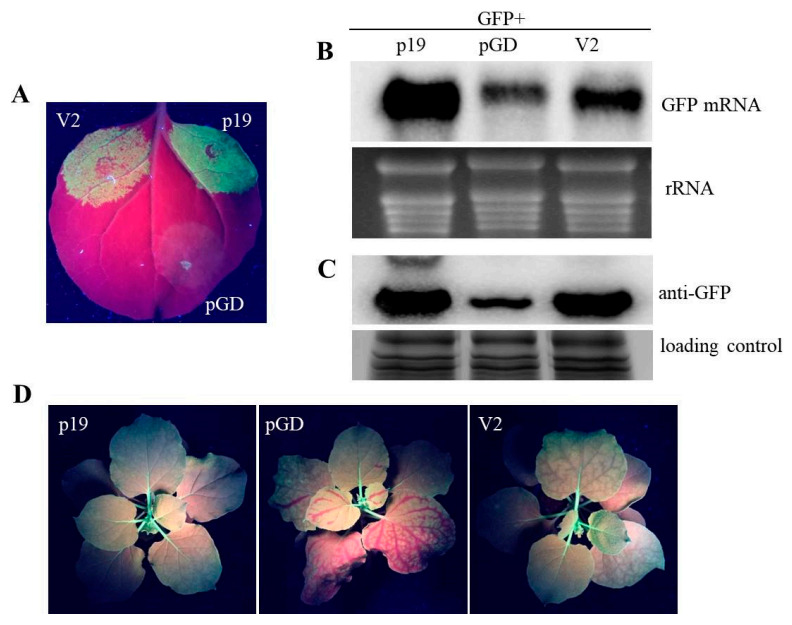
Suppression of posttranscriptional gene silencing by tobacco curly shoot virus (TbCSV) V2. (**A**) Suppression of gene silencing in wild-type *Nicotiana benthamiana* co-agroinfiltrated with *Agrobacterium* strain containing pGD-GFP and a train containing pGD-V2. Co-infiltration with pGD-GFP and pGD or pGD-p19 (expressing p19 protein encoded by TBSV) served as negative or positive control, respectively. Photographs were taken at 3 days post-agroinfiltration (dpa) under long-wavelength UV light. (**B**) Northern blot analysis of green fluorescent protein (GFP) mRNA extracted from the agroinfiltrated leaf regions. Blots were hybridized with probes specific for GFP mRNA. Ethidium-bromide-stained rRNA was used as an RNA loading control. (**C**) Western blot analysis of the GFP protein accumulation in leaf patches with different treatment at 3 dpa. The Coomassie-blue-stained gel served as a loading control. (**D**) TbCSV V2 suppresses systemic GFP silencing triggered by GFP sense RNA. Representative images were taken at 20 dpa. The lower leaves of 16c line *N. benthamiana* were co-infiltrated with *Agrobacterium* strain containing pGD-GFP and a train containing the indicated construct, and the GFP fluorescence on the upper leaves were detected at 20 dpa.

Previous reports showed that V2 encoded by some other genimiviruses function as VSRs [44,51,52]. However, besides TbCSV C2 and C4 proteins whose suppressor activities have been reported, if the V2 protein encoded by TbCSV is the third VSR remains to be determined. In this study, the coding sequence of TbCSV V2 was cloned into the binary vector pGD (pGD-V2) and subjected to *Agrobacterium* co-infiltration assay with pGD-GFP, as described previously, to test its suppression activity [19,53]. Wild-type *Nicotiana benthamiana* leaf patches agroinfiltrated with pGD-V2/pGD-GFP showed obvious GFP fluorescence at 3 days post-agroinfiltration (dpa), with less intensity of the fluorescence than that emanated from the leaf patches co-infiltrated with p19 encoded by tomato bushy stunt virus (TBSV) and GFP (pGD-p19/pGD-GFP), the positive control (Figure 1A). In contrast, there was hardly any GFP fluorescence detected in leaf patches of negative control plants co-infiltrated with pGD-GFP and the empty vector pGD (Figure 1A). Furthermore, the infiltrated leaf patches were subjected to northern blot and western blot analyses to quantify the accumulations of GFP mRNA and protein, and the results showed significantly more GFP mRNA and protein accumulations in pGD-V2/pGD-GFP or pGD-p19/pGD-GFP infiltrated leaf patches than that in the negative control, which were in agreement with the fluorescence levels observed (Figure 1B,C). The results demonstrated that TbCSV V2 can inhibit local RNA silencing triggered by positive-sense GFP RNA. 

To further determine whether V2 could suppress systemic RNA silencing, *Agrobacterium* co-infiltration assay was performed as described above on GFP-expressing transgenic *N. benthamiana* line 16c. At 10 dpa, for 10 of the 12 plants infiltrated with pGD/pGD-GFP, GFP fluorescence began to exhibit loss along the vein of the upper non-infiltrated leaves. In contrast, in 10 of 12 plants treated with pGD-V2/pGD-GFP and all 12 treated plants with pGD-p19/pGD-GFP, the non-infiltrated upper leaves retained green fluorescence at this time point. At 20 dpa, 8 plants infiltrated with pGD-V2/pGD-GFP and 2 plants infiltrated with pGD/pGD-GFP retained GFP fluorescence, respectively, whereas all the positive control plants retained GFP fluorescence (Figure 1D). These results suggested that TbCSV V2 can suppress systemic RNA silencing triggered by positive-sense GFP RNA with a lower activity than the strong VSR p19.

### 2.2. Residues 1–92 Are Necessary for VSR Activity of V2

**Figure 2:**
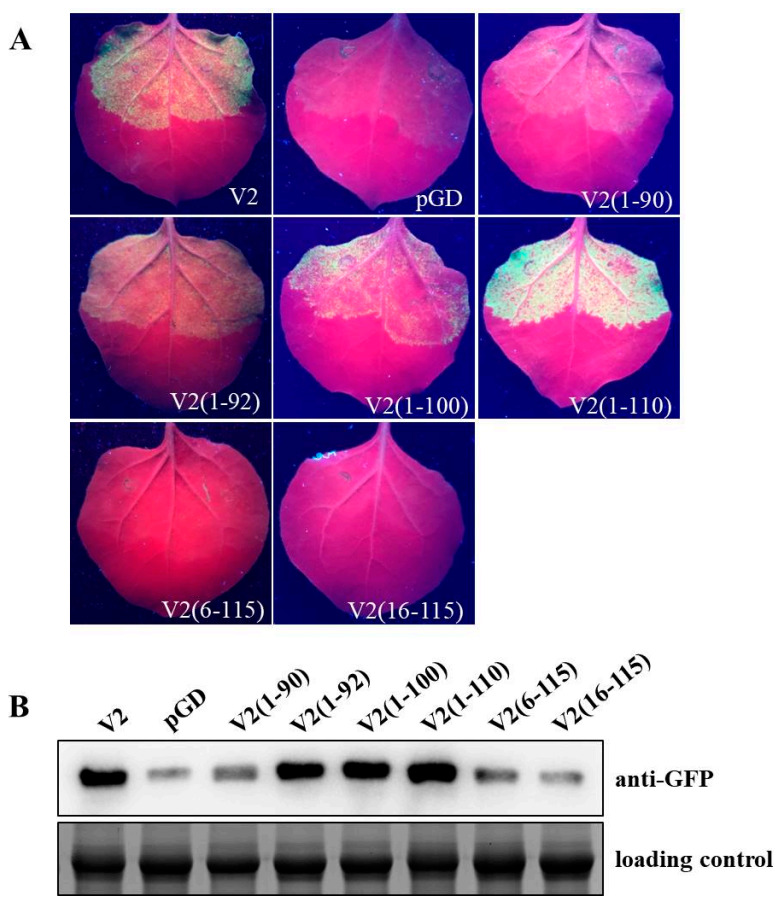
Amino acid region required for V2 suppression of RNA silencing. (**A**) Examination of gene silencing suppression in wild-type *N. benthamiana* leaves co-infiltrated with an *Agrobacterium* strain containing pGD-GFP and a strain containing one of the truncated mutants of V2. Co-infiltration with pGD-GFP and pGD or pGD-V2 served as negative and positive control, respectively. Photographs were taken at 3 dpa. (**B**) Western blot analyses of the GFP protein accumulation in leaf patches with different construct infiltrations at 3 dpa. The Coomassie-blue-stained gel served as loading control.

Protein sequence analyses (http://www.ebi.ac.uk/interpro/) showed that a “Genimi_V2” (amino acids 1–78, aa 1–78) domain and a “WCCH” (aa 79–102) motif are contained in V2 (115 amino acids). Secondary structure analysis (http://bioinf.cs.ucl.ac.uk/psipred/) predicted that V2 possesses six α-helices (aa 13–31, aa 37–50, aa 55–56, aa 73–75, aa 91–92 and aa 103–107) (Figure S1). Based on the results of the analyses, six V2 truncated mutants—V2 (1–110), V2 (1–100), V2 (1–92), V2 (1–90), V2 (16–115) and V2 (6–115)—were expressed by pGD vector. These V2 mutants were subjected to agroinfiltration assay to determine their activities on suppressing local RNA silencing, and pGD-V2 and pGD empty vector were used as positive and negative controls, respectively. At 3 dpa, wild-type *N. benthamiana* leaf patches coinfiltrated with V2 (1–92), V2 (1–100) or V2 (1–110) and pGD-GFP showed GFP fluorescence, suggesting the retainment of RNA silencing suppression activity (Figure 2A). However, there was no GFP fluorescence detected in leaf patches of plants infiltrated with V2 (1–90)/pGD-GFP, V2 (6–115)/pGD-GFP, V2 (16–115)/pGD-GFP or pGD/pGD-GFP combinations (Figure 2A). In western blot assay, obviously higher levels of GFP accumulation could be detected in leaf patches infiltrated with V2(1–110)/pGD-GFP, V2 (1–100)/pGD-GFP, V2 (1–92)/pGD-GFP or V2/pGD-GFP constructs than that treated with V2 (1–90)/pGD-GFP, V2 (6–115)/pGD-GFP, V2 (16–115)/pGD-GFP or pGD/pGD-GFP constructs, which were in agreement with the fluorescence observations (Figure 2B). Furthermore, the multiple amino acid sequences alignment of V2 proteins encoded by TbCSV and some other begomoviruses was performed, and a conserved amino acid region (aa 1–92) and a variable region (aa 93 to the end) among these V2 homologues were presented (Figure S2). Interestingly, the predicted first five α-helices (aa 13–31, aa 37–50, aa 55–56, aa 73–75 and aa 91–92) were located in the conserved amino acid region of V2. These results demonstrated that the phylogenetically conserved first 1–92 amino acid region of the V2 protein, which consists of five putative α-helices, is critical for the local RNA silencing suppression activity.

### 2.3. Basic and Ring-Structured Amino Acids Located in the Conserved Region Were Required for V2 Suppressor Activity

**Figure 3:**
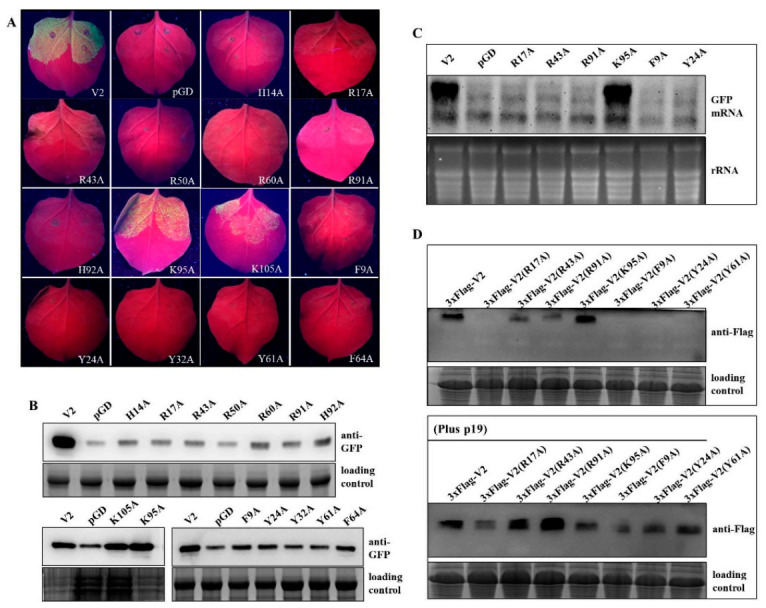
Silencing suppression activity of substitution mutants of V2. (**A**) Co-infiltration of wild-type *N. benthamiana* leaf patches with pGD-GFP and one of the substitution mutants of V2 including H14, R17, R43, R50, R60, R91, H92, K95, K105, F9, Y24, Y32, Y61 and F64. Co-infiltration with pGD-GFP and pGD or pGD-V2 served as negative and positive control, respectively. Photographs were taken at 3 dpa. (**B**) Western blot analyses of the GFP protein accumulation in leaf patches infiltrated with different constructs at 3 dpa. The Coomassie-blue-stained gel served as a loading control. (**C**) Northern blot analysis of green fluorescent protein (GFP) mRNA extracted from the agroinfiltrated regions. Blots were hybridized with probes specific for GFP mRNA. Ethidium-bromide-stained rRNA was used as an RNA loading control. (**D**) Western blot analyses of the accumulation of flag-tagged V2 and V2 mutants. “Plus p19” indicates the presence of tomato bushy stunt virus-encoded p19. The Coomassie-blue-stained gel served as loading control.

Our present work suggested that the α-helices and conserved residues should contribute to the VSR activity of V2 (Figure 2, Figure S2). Interestingly, previous reports revealed that basic residues located in α-helix regions of BYV p21 and GLRaV-2 p24 are critical for RNA silencing suppression [18,19]. To determine whether the conserved basic residues located in the first five α-helix regions are crucial for the RNA silencing suppression activity of V2, the basic residues in α-helix 1 (H14, R17), α-helix 2 (R43, R50), α-helix 3 (R60), α-helix 5 (R91 and H92) and two basic amino acids (K95 and K105) located in the variable region (aa 93–115) were substituted with alanine (A) and subjected to agroinfiltration assays. Wild-type V2 and pGD empty vector served as positive and negative controls, respectively. The results showed that the mutant K95A and K105A could still suppress the positive-sense GFP RNA-triggered local RNA silencing, indicated by the GFP fluorescence similar to that detected on pGD-V2/pGD-GFP infiltrated positive control plants. In contrast, there was hardly any fluorescence detected on leaves co-expressed with H14A, R17A, R43A, R50A, R60A, R91A or H92A and GFP, suggesting these mutants lost the activity to suppress silencing of the exogenous GFP (Figure 3A). Western blot assays were performed to examine the accumulation of GFP, and the blotting results were consistent with the fluorescence observations that obviously decreased GFP protein accumulations were detected in the leaves infiltrated with H14A, R17A, R43A, R50A, R60A, R91A and H92A. In contrast, for K95A and K105A mutants, the GFP protein level was almost comparable with that in wt V2-infiltrated plants (Figure 3B). These results indicated that the evolutionarily conserved basic residues located in α-helix regions are crucial for the RNA silencing suppression activity of V2.

Ring-structured amino acids were reported to affect the VSR activity of melon aphid-borne yellows virus (MABYV) p0 and GLRDV-2 p24 proteins with unknown precise mechanism [19,54]. We generated five substitutions for amino acids with a ring-structured side chain located in the conserved region (F9A, Y24A, Y32A, Y61A and F64A). Agroinfiltration assays were conducted to investigate the suppressor activity of these mutants, and GFP fluorescence on leaves co-infiltrated with GFP and F9A, Y24A, Y32A, Y61A or F64A were not easily detectable, suggesting that five mutants failed to suppress RNA silencing (Figure 3A). Consistent with the results, Western blotting showed obviously decreased levels of GFP protein accumulations in leaf patches co-infiltrated with GFP and F9A, Y24A, Y32A, Y61A, F64A or pGD empty vector (Figure 3B). These results suggest that these five conserved ring-structured amino acids at positions 9, 24, 32, 61 and 64 are crucial for the RNA silencing suppression activity of V2.

Northern blot analysis was performed to determine the GFP mRNA accumulations in leaf patches agroinfiltrated with V2 mutants (R17A, R43A, R91A, K95A, F9A and Y24A). Our results showed that obviously decreased GFP mRNA levels were detected in leaf patches co-infiltrated with GFP and R17A, R43A, R91A, F9A, Y24A or pGD empty vector. In contrast, for the K95A mutant, which retains the RNA silencing suppression activity, the GFP mRNA level was comparable with that in the wt V2-infiltrated plant (Figure 3C). 

Seven V2 mutants were subjected to Western blot analyses for assessing the protein accumulations. First, wt V2 and V2 mutants were tagged with a 3xFlag epitope at their N-termini, and the effects of the tag on RNA silencing suppression activity of V2 were investigated. Agroinfiltration assay indicated that 3xFlag-V2 can still suppress positive-sense GFP RNA triggered gene silencing, although with decreased activity (Figure S3). Subsequently, the protein accumulation levels of V2 mutant were examined at 3 dpa in the presence or absence of TBSV p19. The protein accumulation levels of R43A and R91A were lower than that of wt V2 in the absence of p19, whereas levels comparable with those of wt V2 and K95A, a mutant that retains the suppressor activity, were reached in the presence of p19 (Figure 3D). We noticed that the accumulation level of R91A increased markedly in the presence of p19, which was even higher than that of wt V2. For V2 mutants R17A, F9A, Y24A and Y61A, the protein accumulations were hardly detected in the absence of p19. However, p19 can indeed increase the accumulation levels of these mutants (Figure 3D). These results suggested that the decreased accumulations of V2 mutants can be attributed, at least in part, to the robust RNA silencing mechanism. 

### 2.4. Specific Amino Acids with Positive Charge or Those Required for V2 Self-Interaction Are Critical for RNA Silencing Suppression

**Figure 4:**
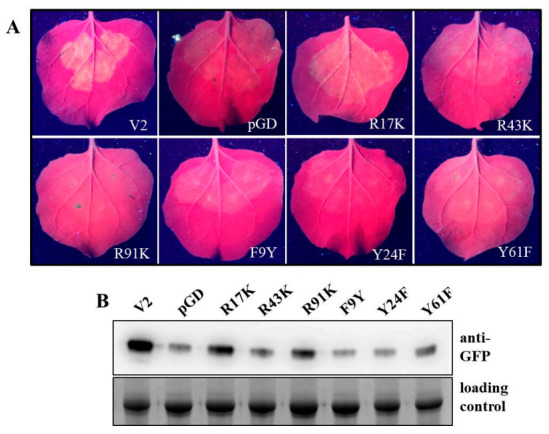
Silencing suppression activity of V2 mutants substituted with isomorphic amino acids. (**A**) Co-infiltration of wild-type *N. benthamiana* leaf patches with pGD-GFP and one of the substitution mutants of V2 including R17K, R43K, R91K, F9Y, Y24F and Y61F. Co-infiltration with pGD-GFP and pGD or pGD-V2 served as negative and positive control, respectively. Photographs were taken at 3 dpa. (**B**), Western blot analysis of the GFP protein accumulation in agroinfiltrated leaf patches with different constructs at 3 dpa. The Coomassie-blue-stained gel served as a loading control.

Similarly, we created mutations targeting basic or ring-structured amino acids by substituting with isomorphic amino acids. Three basic amino acids (R17, R43 and R91) and three amino acids with a ring-structured side chain (F9 with a benzene ring, Y24 and Y61 with phenol ring) located in conserved motifs were selected for further analyses. We substituted the R17, R43 and R91 with lysine (K), another positively charged residue, respectively. Similarly, we generated substitutions targeting F9, Y24 and Y61 with tyrosine (Y), phenylalanine (F) and F, respectively. Agroinfiltration assays showed that leaves co-infiltrated with GFP and R17K displayed weaker GFP fluorescence than that of leaves co-infiltrated with pGD-GFP/pGD-V2. However, fluorescence in leaves co-infiltrated with R43K, R91K, F9Y, Y24F or Y61F and GFP was not easily detectable (Figure 4A). Western blotting confirmed the fluorescence observations results (Figure 4B). These results suggest that basic residue R17 can be replaced by K, with attenuated RNA silencing suppression activity. In contrast, R43, R91, F9, Y24 and Y61 could not be replaced by residues with similar properties for recovering V2′s suppressor activity.

**Figure 5:**
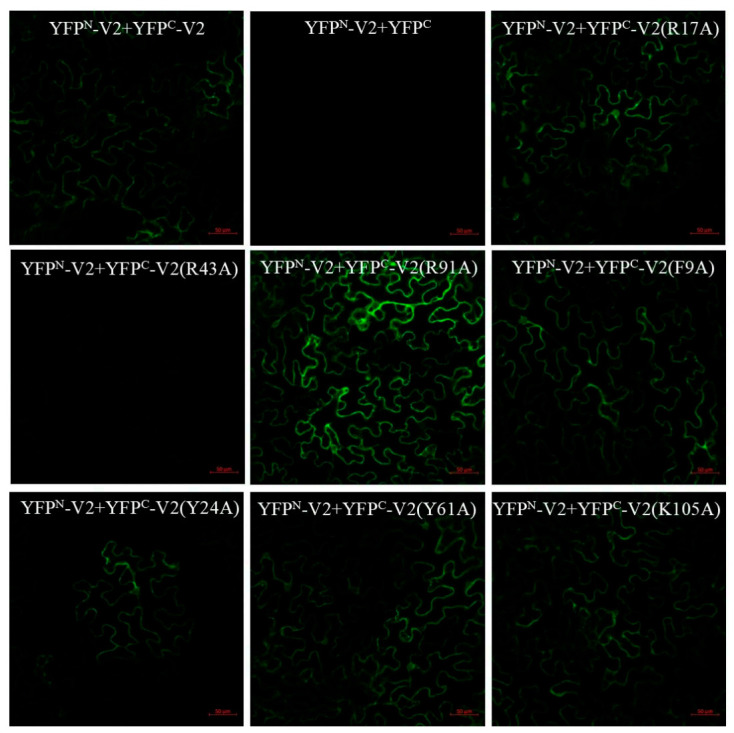
Effects of amino acid substitutions on V2 self-interaction. Bimolecular fluorescence complementation (BiFC) analyses of the interaction between wt V2 and V2 mutants in *N. benthamiana*. Different BiFC vector combinations were co-agroinfiltrated into *N. benthamiana* leaves, respectively, and YFP fluorescence were detected at 3 days post-agroinfiltration (dpa) at wavelength of 488 nm. Bars = 50 μm.

A previous report revealed that the self-interaction of TYLCV V2 is associated with its biological properties, including subcellular localization and pathogenicity [55]. In our report, we generated a series of constructs expressing wt V2 (YFP^N^-V2 and YFP^C^-V2) or V2 mutants (YFP^C^-V2(R17A), YFP^C^-V2(R43A), YFP^C^-V2(R91A), YFP^C^-V2(F9A), YFP^C^-V2(Y24A), YFP^C^-V2(Y61A) and YFP^C^-V2(K105A)) for bimolecular fluorescence complementation (BiFC) analyses. GFP fluorescence could be observed in YFP^N^-V2/YFP^C^-V2 co-expressed *N. benthamiana* cells, suggesting the self-interaction of TbCSV V2 in vivo (Figure 5). In contrast, no fluorescence could be detected in YFP^N^-V2/YFP^C^ pair expressed cells. The lack of VSR activities could lead to decreased mRNA and protein accumulation levels of V2 mutants because of the robust RNA silencing mechanism, and ultimately affect the V2 mutants’ self-interactions [56]. To exclude this potential effect, we examined the self-interaction between V2 mutants and wt V2 in *N. benthamiana* leaf epidermic cells by BiFC. In this study, reconstitution of YFP fluorescence in *N. benthamiana* cells co-infiltrated with YFP^N^-V2/YFP^C^-V2 (R17A), YFP^N^-V2/YFP^C^-V2 (R91A), YFP^N^-V2/YFP^C^-V2 (F9A), YFP^N^-V2/YFP^C^-V2(Y24A), YFP^N^-V2/YFP^C^-V2(Y61A) and YFP^N^-V2/YFP^C^-V2(K105A) could be observed, just as that shown in YFP^N^-V2/YFP^C^-V2 co-expressed cells. However, co-infiltration with YFP^N^-V2/YFP^C^-V2 (R43A) led to an obviously weaker YFP signal, suggesting that the arginine (R) at the position 43 is critical for the self-interaction of V2 in plants (Figure 5). 

Taken together, among the conserved basic or ring-structured amino acids that are necessary for the VSR activity of V2, R17 is partially dependent on its positively charged property, which might function in RNA binding during RNA silencing suppression, whereas R43 is critical for self-interaction and RNA silencing suppression of TbCSV V2.

### 2.5. The Suppressor Activity of V2 Is Dispensable for Symptom Aggravation of Heterogenous PVX Infection

**Figure 6:**
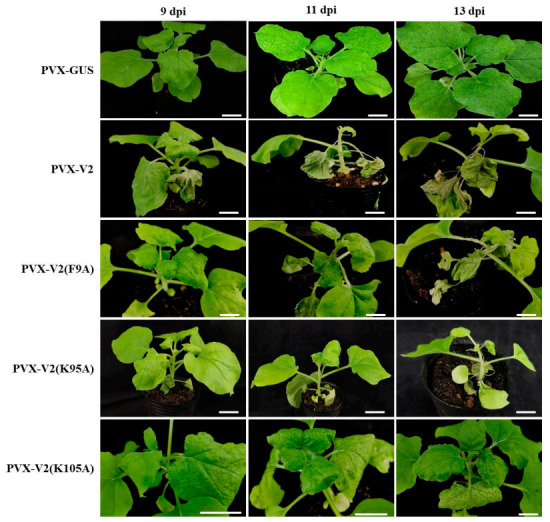
Symptom aggravation induced by V2-expressing PVX is independent of the RNA silencing suppression activity of V2. Symptoms induced by chimeric PVX expressing wt V2 or V2 mutants. PVX expressing partial *β-glucuronidase* (GUS) gene served as control. PVX-V2, PVX-GUS, PVX-V2(F9A), PVX-V2(K95A) and PVX-V2(K105A) were inoculated into *N. benthamiana* respectively at the six-leaf stage, and symptom images were taken at 9, 11 and 13 days post inoculation (dpi). Scale bar, 3 cm.

Some reports showed that V2 proteins encoded by some geminiviruses could substantially function in viral pathogenicity [43,55,57,58,59]. To investigate the role of V2 encoded by TbCSV in virus infection, V2 coding sequence was inserted into and heteroexpressed by potato virus X. The recombinant infectious clone named PVX-V2 was agroinfiltrated into *N. benthamiana* at the six-leaf stage, and the hypersensitive response began to emerge on the infiltrated leaves at 5 days postinoculation (dpi) (data not shown). At 9 dpi, the cell death phenotype could be obviously observed on the whole infiltrated leaves, and subsequently, the cell death extended to upper systemically infected leaves until systemic necrosis occurred (Figure 6). In contrast, only chlorosis and mosaic phenotype could be observed on the systemically infected leaves of PVX-inoculated control plants during infection. Our results suggest that TbCSV V2 can exacerbate virus infection and induce cell death response. 

It has been reported that co-expression of PVX and RNA silencing suppressors, Hc-Pro encoded by plum pox virus (PPV) or p19 encoded by tomato bushy stunt virus (TBSV), can induce hypersensitive response-like necrosis of *N. benthaniana* leaves [60]. As a VSR, when V2 was heterogenously expressed by PVX, the tissue necrosis could be induced (Figure 6). Thus, it is necessary to determine whether the suppressor activity of V2 is responsible for the severe symptom induction. For this purpose, the amino acid point mutants of V2, including suppression activity retaining mutant V2-K95A and V2-K105A and inactivated V2-F9A, were used to be expressed by PVX. At 9 dpi, no visible necrosis phenotype could be detected on PVX-V2(F9A)-, PVX-V2(K95A)- or PVX-V2(K105A)-infected *N. benthamiana* plants, as that shown on PVX-GUS-inoculated negative control plants. At 11 dpi, systemic necrosis occurred on the leaves of PVX-V2-, PVX-V2(F9A)- and PVX-V2(K95A)-infected plants, whereas only mild local necrosis spots emerged on PVX-V2(K105A)-inoculated plants. Furthermore, necrosis on some of the petioles and leaves developed for PVX-V2-, PVX-V2(F9A)- and PVX-V2(K95A)-infected plants at 13 dpi. In contrast, there were still only denser local necrosis spots and chlorosis on the upper leaves of PVX-V2(K105A)-infected plants (Figure 6). These results indicated that the mutation of K105A attenuated the activity of V2 to induce tissue necrosis when expressed by PVX. However, V2-F9A and V2-K95A could both induce necrosis, although the V2-F9A mutant lost the RNA silencing suppression activity, suggesting that the symptom aggravation activity of V2 does not depend on the suppressor activity. Furthermore, the relative accumulations of PVX RNA in PVX-V2- or PVX-V2-mutant-inoculated plants compared with PVX-GUS-inoculated plants were determined by RT-qPCR, and the results showed that the PVX RNA accumulation levels were not significantly affected by V2′s suppressor activity at 9, 11 and 13 dpi (Figure S4). 

Taken together, PVX-mediated heterogenous expression of TbCSV V2 can aggravate the symptoms, ultimately leading to systemic necrosis of infected *N. benthamiana* plants, and this function of V2 is independent of its suppressor activity as well as the increased accumulation of PVX.

### 2.6. V2 Is Critical for TbCSV Infection and Symptom Development

**Figure 7:**
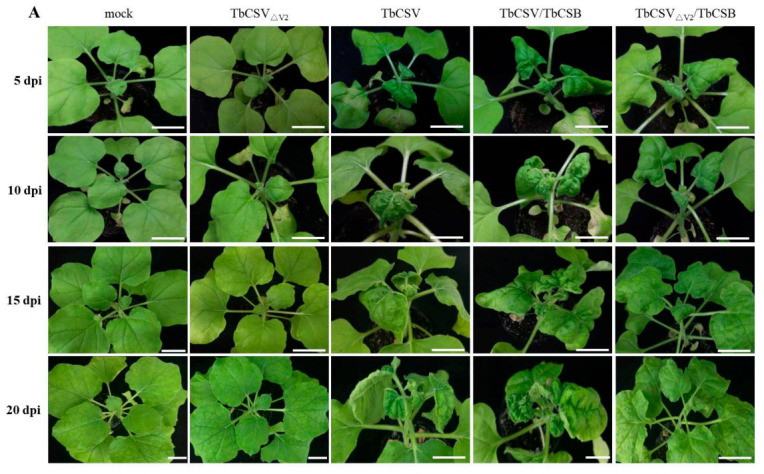

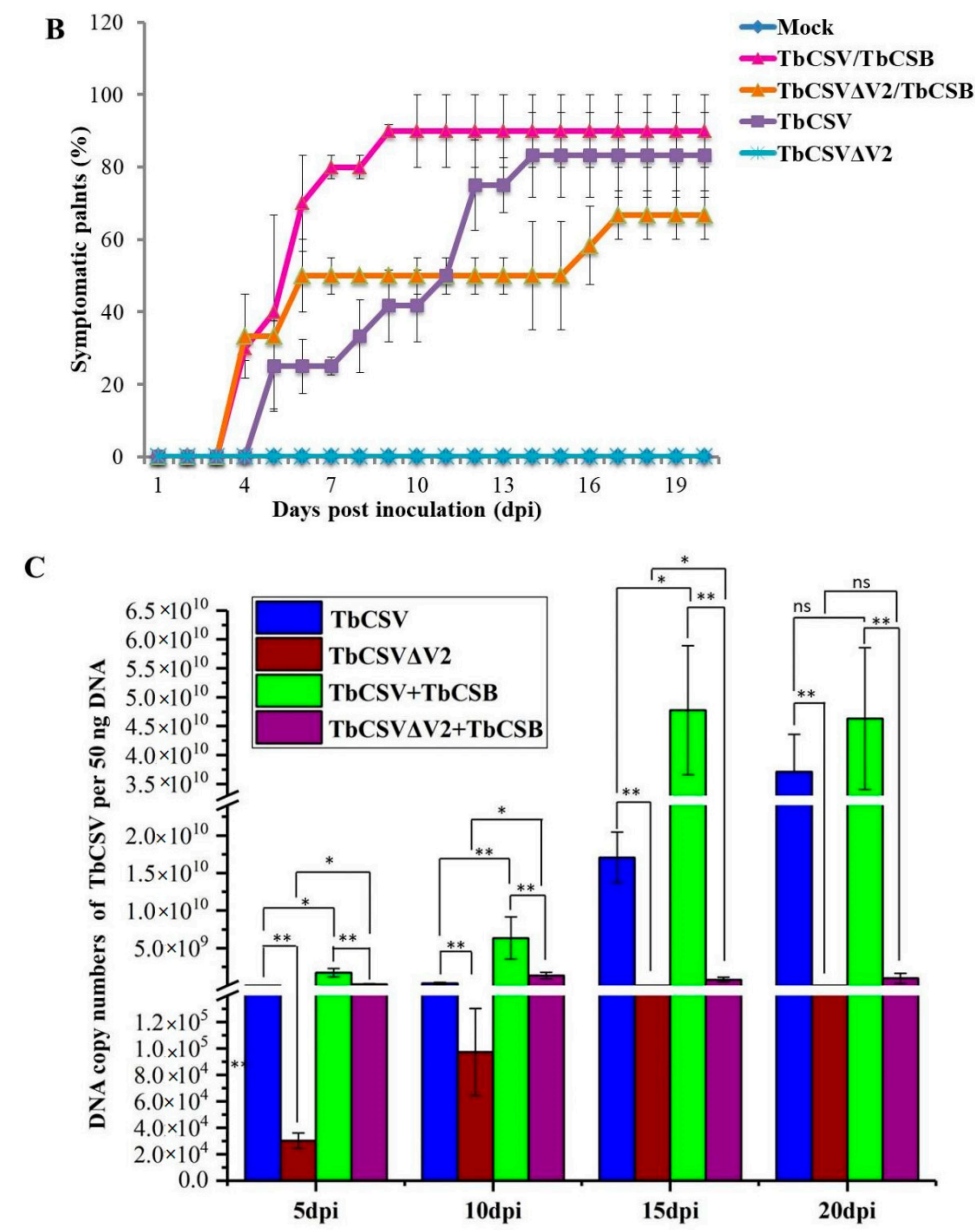
Disease symptoms and virus accumulations in the TbCSV-, TbCSV_ΔV2_-, TbCSV/TbCSB- or TbCSV_ΔV2_/TbCSB-infected *N. benthamiana* plants. (**A**) Symptoms induced by TbCSV-, TbCSV_ΔV2_-, TbCSV/TbCSB- or TbCSV_ΔV2_/TbCSB inoculations at 5, 10, 15 and 20 days postinoculation (dpi). Buffer inoculation (mock) served as a negative control. Scale bar, 3 cm. (**B**) The incidence of appearance of abnormal phenotypes on the upper leaves at different time points. (**C**) Accumulation of TbCSV DNA in systemically infected leaves of TbCSV-, TbCSV_ΔV2_-, TbCSV/TbCSB- or TbCSV_ΔV2_/TbCSB-inoculated *N. benthamiana* plants at different time points. Quantitative PCR analyses of TbCSV copy numbers in systemically infected leaves at 5, 10, 15 and 20 dpi. The experiments were conducted three times independently, and at least three technical replicates were used each time. Error bars represented the means ± SE. Significant differences were indicated using Student’s *t* test: ** indicates *p* < 0.01, * indicates *p* < 0.05, “ns” indicates no significant difference. *N. benthamiana* 25S ribosomal RNA was used as the internal control.

To further reveal the functions of V2 on TbCSV infection, viral mutant infectious clone with untranslated V2 (designated as TbCSV_∆V2_) was constructed by changing the start codon of ATG to ATC. Subsequently, TbCSV, TbCSV_∆V2_, TbCSV associated with DNAβ satellite (TbCSV/TbCSB) and TbCSV_∆V2_/TbCSB were inoculated into wild-type *N. benthamiana* plants using the method previously reported, and inoculation with buffer (mock) served as a control [33]. At 5 dpi, an obvious downward curling phenotype of the upper leaves emerged on TbCSV-inoculated plants, and the symptoms including leaf curling and shrinking became more obvious over time (Figure 7A). The symptoms could be observed on 80 percent plants inoculated by TbCSV at 14 dpi. In contrast, TbCSV_∆V2_ failed to induce visible symptoms for all the inoculated plants until 20 dpi, as that shown on mock-inoculated control plants (Figure 7A,B). For TbCSV/TbCSB inoculation, disease symptoms started to appear at 3 to 4 dpi, with about 90 percent of plants displaying typical symptoms at 8 to 10 dpi. For the plants inoculated with TbCSV_∆V2_/TbCSB, leaf curling and mild shrinking could be observed at 5 and 10 dpi. Interestingly, however, the symptom recovery occurred at 15 and 20 dpi, and obviously less symptomatic plants could be observed (Figure 7A,B). We further determined the virus accumulation levels in systemically infected leaves of these plants with different inoculation treatments using quantitative PCR (qPCR). As shown in Figure 7C, accumulation of TbCSV was significantly lower in plants inoculated with TbCSV_∆V2_ or TbCSV_∆V2_/TbCSB than that detected in plants infected with wild-type TbCSV or TbCSV/TbCSB, respectively. In addition, we also noticed that coinfection with DNAβ could elevate the accumulation of TbCSV_∆V2_ at 5, 10 and 15 dpi. These results suggest that TbCSV V2 can function as a positive regulator of TbCSV infection.

## 3. Discussion

RNA silencing is a conserved antiviral immune mechanism of plants, and encoding one or more RNA silencing suppressors to counter this defense mechanism is a usual strategy recruited by viruses for successful infections [1,61]. In this study, canonical *Agrobacterium* co-infiltration assays were performed to show that TbCSV V2 can suppress positive-sense GFP RNA-induced local and systemic RNA silencing (Figure 1). V2 encoded by TYLCV (without a satellite for infection) has been reported to be a suppressor of TGS and local PTGS, whereas it can only delay rather than block systemic gene silencing completely [46,47,48]. It has been reported that TYLCCNV (with a satellite for infection) encoded V2 protein can suppress local and systemic PTGS, while V2-encoding TYLCCNV infection alone was found to be ineffective for TGS suppression, although whether TYLCCNV V2 itself could inhibit TGS had not yet been investigated [51,62]. We analyzed the amino acid sequences homology of TbCSV V2 and other monopartite begomoviruses encoded V2 proteins whose suppressor activities have been investigated, including proteins derived from TYLCV, TYLCCNV, cotton leaf curl Multan virus (CLCuMuV), CLCuKoV, ToLCJV, ageratum yellow vein virus (AYVV) and WmCSV. The results showed that, except for the high sequence similarity between V2 proteins encoded by TbCSV and CLCuKoV (93.9%), the sequences similarity between TbCSV V2 and other V2 proteins is from 70.4% to 76.5% (Table S1). Previous report showed that CLCuKoV V2 could not suppress TGS [63]. However, whether TbCSV V2 can suppress TGS needs to be experimentally investigated. 

As reported previously on the mechanisms exploited by various VSRs, binding of ds-siRNA is a general strategy for countering antiviral RNA silencing [64,65,66]. For begomoviruses, TYLCV-Is-encoded V2 can bind dsRNA in vitro [67]. Prokaryotic expressed and purified V2 protein of CLCuMuV can preferentially bind double-stranded long RNAs, whereas it failed to bind single-stranded or double-stranded siRNAs [50]. TYLCCNV V2 was reported to bind 21- and 24-nt siRNA duplexes and 24-nt ss-siRNA in electrophoresis mobility shift assays [51]. In our report, the predicted α-helices are required for the RNA silencing suppression activity of TbCSV V2 (Figure 2). It has been reported that tomato aspermy virus 2b adopts the α-helix structures to bind and monitor the preferential size of siRNA duplex for selectively sequestering [68]. The α-helices and the inner basic amino acids of BYV p21 are responsible for functional RNA binding surface formation [18]. Notably, substitution of the conserved basic amino acids R17 with nonpolar alanine abolished the RNA silencing suppression activity of V2, whereas with positively charged lysine (K), VSR activity was partially recovered (Figure 3). These results suggested that RNAs binding may be one of the strategies used by TbCSV V2 to suppress RNA silencing; however, the long or small RNAs binding activity and the binding preference of strand forms have yet to be further investigated. 

Previous reports showed a usual but not inevitable phenotype: ectopic expression of geminiviruses-encoded V2 from a PVX-based vector accelerated local HR-like response and systemic necrosis (SN) in *N. benthamiana*. For instance, the ability of geminiviruses-encoded V2 protein to induce HR and SN has been reported for PaLCuV, CLCuMuV, Tomato leaf curl Java virus-A (ToLCJV-A) and several others [44,45,46,50,51]. In contrast, expression of V2 encoded by EACMCV and ACMV did not induce HR response in *N. benthamiana* [50,57]. In this study, PVX-mediated expression of TbCSV V2 can induce HR-like response and SN phenotype in *N. benthamiana* (Figure 6). However, TbCSV infection alone or associated with TBCSB did not induce visible cell death symptoms, and whether TbCSV C2 functioned in this process as that described previously for PaLCuV needs to be determined [45]. When TbCSV V2 was transiently expressed by a noninfectious binary vector pGD, there was no HR response phenotype observed on the infiltrated leaf patches. It has been reported that PVX-mediated expression of some VSRs could induce HR and SN responses for which PVX P25 is a main determinant [60]. However, the expression of C4 protein, a VSR encoded by malvastrum yellow vein virus, can not induce HR-like cell death [38]. Interestingly, TbCSV V2 mutant with no suppressor activity retained the function to induce HR and SN in *N. benthamiana* (Figure 6). Thus, the HR and SN induction ability should be an intrinsic function of TbCSV V2 for which the suppressor activity is dispensable. Furthermore, the mechanisms underlying the cell death induction by TbCSV V2 and the absence of this phenotype during TbCSV infection remain to be investigated. 

In our report, infection with V2-mutated TbCSV (TbCSV_∆V2_) alone could not induce any symptom on *N. benthamiana*, whereas coinfection with its satellite TbCSB could significantly increase the virus accumulation and induce typical downward curling symptom on upper new leaves at an early stage. However, the symptom was gradually recovered over time, the leaf curling symptom disappeared and only mild leaf abnormality could be observed at 20 dpi (Figure 7). In contrast, *N. benthamiana* plants infected with wild-type TbCSV alone or TbCSV/TbCSB showed a steady increase in symptom severity during the disease process. Symptom recovery is regarded as a virus exclusion phenomenon in newly emerging leaves, which depends on the robust RNA silencing antiviral mechanism in shoot apical meristems (SAM) [69,70]. During this process, the virus-infected plants can develop obvious viral symptoms initially; however, the abnormal phenotypes are then attenuated significantly or even disappear on the upper new leaves, and also the virus accumulation also decreases [1,71]. VSRs are known to be exploited by most viruses to counter RNA silencing defense of hosts for successful infections, such as CMV-encoded 2b and TRV 16K protein, which are responsible for SAM invasion by suppressing RNA silencing [11,72,73,74]. Symptom recovery mechanisms have been investigated for some geminiviruses. For instance, beet curly top virus (BCTV) infection could induce continuous symptoms; however, L2- or L3-mutated virus-inoculated plants displayed a symptom recovery phenotype [75]. Further reports showed that AGO4, DRB3 and DCL3 are involved in symptom remission in BCTV_∆L2_-infected *Arabidopsis*, and DNA methylation-mediated TGS plays an important role in the recovery phenotype [76,77,78]. TbCSV V2 was shown to be a suppressor of PTGS; whether V2-deficiency-induced symptom recovery closely related to the loss-of-suppressor function of V2 remains to be determined. Furthermore, except for V2, C2 and C4 proteins encoded by TbCSV and also βC1 encoded by TbCSB have been confirmed to be suppressors of PTGS [31,32]. However, these three proteins could not effectively complement the V2 function for virus accumulation and induction of continuous symptoms. Thus, it is interesting to determine whether V2 and TbCSV encoded other suppressors act at different infection stages or function with different manners for RNA silencing suppression. 

## 4. Materials and Methods 

### 4.1. Plant Materials and Growth Conditions

Wild-type and GFP-transgenic16c line *N. benthamiana* plants were grown in a growth room at 24 °C under a 16 h/8 h (light/dark) photoperiod cycle.

### 4.2. Plasmid Construction

To identify the RNA silencing suppression activity of TbCSV V2 by agroinfiltration assay, the coding sequences of V2 were amplified from TbCSV-Y35 (GenBank No. AJ420318) isolate infectious clone and inserted into the BglII-HindIII site of binary expression vector pGD to produce pGD-V2 recombinant construct. The truncated derivatives of V2, including V2 (1–90) (contains amino acids 1–90), V2 (1–92), V2 (1–100), V2 (1–110), V2 (6–115) and V2 (16–115), were amplified using pGD-V2 as template and inserted into the BglII-HindIII site of the pGD vector, respectively. To produce V2 substitution mutants, the V2 coding sequence was inserted into pMD19-Tsimple vector and used as a template to amplify the mutated V2 sequence by reverse PCR as described previously [19]. The V2 mutants H14A, R17A, R43A, R50A, R60A, R91A, H92A, K95A, K105A, F9A, Y24A, Y32A, Y61A and F64A were digested and cloned into the BglII-HindIII site of pGD. 

To test the pathogenicity of TbCSV V2 or V2 mutants, the coding sequences were amplified from pGD-V2, pGD-V2-F9A, pGD-V2-K95A and pGD-V2-K105A, respectively, using a primer pair (PVX-V2-F/PVX-V2-R) and subcloned into the ClaI-SalI site of pGR106 [51]. 

To examine the self-interaction of V2, the coding sequences were amplified from pGD-V2 using primer pairs YFPN-V2-F/YFPN-V2-R and YFPC-V2-F/YFPC-V2-R, and subcloned into N-terminus of YFP and C-terminus of YFP respectively. To examine the self-interaction of V2 mutants, the coding sequences of V2 mutants were amplified from pGD-V2-R17A, pGD-V2-R43A, pGD-V2-R91A, pGD-V2-F9A, pGD-V2-Y24A, pGD-V2-Y61A and pGD-V2-K105A using primer pair YFPC-V2-F/YFPC-V2-R and cloned into C-terminus of YFP, respectively. 

To obtain TbCSV V2 mutant infections clone in which potential start codons of V2 were substituted, a construct including tandem 1 and 0.9 copy of TbCSV-Y35 clone was used as the template for PCR. First, a fragment with mutations of the second ATG codon of the first 1 copy and the first ATG codon of the second 0.9 copy was amplified using ΔV2-F1/ΔV2-R1 primer pair. The product was extended using ΔV2-F2/ΔV2-R1 and ΔV2-F3/ΔV2-R1 primer pairs in turn, to produce a fragment designated as P1 with an extension at 5′ terminus overlapping with pBinPLUS vector. Primer pair ΔV2-F1/ΔV2-R2 was used to amplify a fragment with mutation to another potential ATG codon of the second 0.9 copy. Subsequently, the products were extended by PCR, using ΔV2-F2/ΔV2-R2 and ΔV2-F3/ΔV2-R2 primer pairs in turn to produce P2 fragment overlapping with P1 at 5′ terminus and with pBinPLUS vector at 3′ terminus. Eventually, P1 and P2 were inserted into pBinPLUS vector that digested with BamHI-HindIII enzymes, by the homologues recombination method, to produce the TbCSVΔV2 construct for further use.

### 4.3. Agroinfiltration and GFP Fluorescence Imaging

For local and systemic RNA silencing suppression activity identification, each plasmid that needed to be tested was transformed into *Agrobacterium tumefaciens* GV3101, and subsequent infiltration and fluorescence detecting were performed as described previously [19,79].

### 4.4. Western Blot Analyses

The protein extraction from *N. benthamiana* leaves and Western blot assay were performed as described previously [38]. Briefly, total protein was extracted from infiltrated *N. benthamiana* leaf patches using 2xSDS sample buffer and boiled at 100 °C for denaturation. The protein samples were separated by 10% sodium dodecylsulfate-polyacrylamide gel electrophoresis (SDS-PAGE), and an anti-GFP polyclonal antibody (CWBIO, Beijing, China) was used to probe the blots, and then incubating with a horseradish peroxidase (HRP)-conjugated goat antirabbit IgG secondary antibody (CWBIO, Beijing, China). Finally, GFP signal was detected with the Super ECL plus Western blotting kit according to the manufacturer’s instructions (BIOGROUND, Chongqing, China).

### 4.5. RNA Extraction and Northern Blot Analyses

Total RNA was extracted from the agroinfiltrated leaf patches of *N. benthamiana* plants using TRIzol reagent (Invitrogen, Carlsbad, CA, USA) as instructed by the manufacturer. For accumulation levels analyses of GFP mRNA, probes were prepared using a PCR DIG probe synthesis kit (Roche, Mannheim, Germany) using specific primers (Table S2). Total RNAs were quantified and 15 µg of RNA samples were subjected to electrophoresis using a 1.2% denaturing agarose gel; then, they were transferred to Hybond N+ nylon membranes (GE Healthcare, Buckinghamshire, UK http://www.gelifesciences.com.cn/CNLS/indexAction.action). The hybridization was performed using a DIG-High Prime DNA labeling and detection starter kit II (Roche) according to the manufacturer’s instructions. GFP mRNA signal was detected using the ChemiDoc™ Touch Imaging System (Bio-Rad, Hercules, CA, USA).

### 4.6. BiFC Assay

Plasmids YFPN-V2, YFPC-V2, YFPC, YFPC-V2(R17A), YFPC-V2(R43A), YFPC-V2(R91A), YFPC-V2(F9A), YFPC-V2(Y24A), YFPC-V2(Y61A) and YFPC-V2(K105A) were transformed into *Agrobacterium tumefaciens* strain GV3101, respectively. The cultured *Agrobacterium* cells were resuspended in MMA buffer (10 mmol/L MES/NaOH, pH 5.6, 10 mmol/L MgCl2, 150 µmol/L acetosyringone) to an OD600 of 0.5, and YFP split combinations were prepared for coinfiltration. At 3 dpa, the fluorescence in agroinfiltrated leaves were detected under a confocal laser scanning microscope (LSM780, Carl Zeiss, Oberkochen, Germany) at a wavelength of 488 nm. Experiments were repeated three times.

### 4.7. qPCR and qRT-PCR Analysis

For quantitative analyses of PVX RNA accumulation, SYBR Green real-time RT-PCR was performed using the reagent and method as described previously with the primer pair qPVX-F/qPVX-R [33]. The expression level of the *N. benthamiana* 25S ribosomal RNA gene (Accession no. KP824745) was examined with primer pair qNbrRNA-F/qNbrRNA-R and used as the internal control (Table S2) [33,80,81]. The relative expression levels of target genes were calculated using the 2^−∆∆CT^ method [82]. 

To determine the accumulation of TbCSV, the standard curve for estimating the TbCSV DNA copy number was established first. The full-length TbCSV sequence was inserted into the pMD19-T vector to generate the pMD-TbCSV plasmid, followed by a tenfold serial dilution of the plasmid from 101 to 108, which were used as templates for qPCR with NovoStart SYBR qPCR SuperMix Plus kit according to the manufacturer’s instructions (Novoprotein, Shanghai, China), using primers specific to TbCSV V1 gene (qTbCSV-F/qTbCSV-R). An optimal standard curve for TbCSV with a coefficient of regression *R*^2^ = 0.990 was obtained by optimizing the primer concentration in reactions. To calculate TbCSV copy number based on the Ct values generated from qPCR assay, DNA was extracted from the upper leaves of infected *N. benthamiana* plants using the CTAB method, and then was adjusted to 50 ng before it was used as a template for qPCR. Three independent experiments were conducted with at least three biological replicates for each treatment. The results shown are the means from three individual experiments. Significant differences were indicated using Student’s *t* test: * indicates *p* < 0.05; ** indicates *p* < 0.01. 

The constructs and primers used in this study are listed in Supplemental Table S2.

## 5. Conclusions

To better understand the function of TbCSV V2 during viral infection, in the present study, the inoculation assay using TbCSV infectious clone mutant with untranslated V2 showed that V2 protein is critical for viral infection and continuous symptom development. Agroinfiltration assays indicated that V2 can suppress local and systemic gene silencing. Deletion and site-directed substitutions analyses demonstrated that the α-helix regions and the conserved basic or ring-structured amino acids are critical for RNA silencing suppression activity of V2. Further analyses suggested that RNA binding and self-interaction of V2 may contribute to its suppressor activity. As that shown for some other homologues, TbCSV V2 can also exacerbate the symptoms induced by PVX infection, and we first confirmed that this function of V2 is independent of its suppressor activity.

## Data Availability

Please refer to suggested Data Availability Statements in section “MDPI Research Data Policies” at https://www.mdpi.com/ethics.

## Supplementary Materials

Supplementary materials can be found at https://www.mdpi.com/1422-0067/22/2/923/s1. Figure S1: The predicted secondary structure of TbCSV V2. The red asterisks indicate the amino acids subjected to substitutions; Figure S2: The multiple alignments of the V2 protein sequences of several geminiviruses; Figure S3: Suppression of local silencing by flag tagged TbCSV V2 protein. Figure S4: RT-qPCR analyses of PVX relative accumulation levels in V2 or V2 mutants expressed N. benthamiana plants at 9, 11 and 13 dpi. Each group contains 6 plants and these experiments were repeated at least twice. Error bars represented the means±SE. N. benthamiana 25S rRNA gene was used as the internal control; Table S1. Homology of V2 proteins encoded by 8 geminiviruses; Supplemental Table S2. Constructs and primers used in this study.

## Abbreviations

TbCSV	tobacco curly shoot virus
TbCSB	betasatellite of TbCSV
PV	potato virus X
TBSV	tomato bushy stunt virus
VSR	viral suppressor of RNA silencing
dpi	days postinoculation
dpa	days postagroinfiltration
GFP	green fluorescence protein

## References

[1] Baulcombe D. (2004). RNA silencing in plants. Nature.

[2] Carbonell A., Carrington J.C. (2015). Antiviral roles of plant ARGONAUTES. Curr. Opin. Plant Biol..

[3] Ding S.W., Lu R. (2011). Virus-derived siRNAs and piRNAs in immunity and pathogenesis. Curr. Opin. Virol..

[4] Zhang C., Wu Z., Li Y., Wu J. (2015). Biogenesis, function, and applications of virus-derived small RNAs in plants. Front Microbiol..

[5] Smith N.A., Eamens A.L., Wang M.B. (2011). Viral small interfering RNAs target host genes to mediate disease symptoms in plants. PLoS Pathog..

[6] Yang Z., Li Y. (2018). Dissection of RNAi-based antiviral immunity in plants. Curr. Opin. Virol..

[7] Ding S.W., Voinnet O. (2007). Antiviral immunity directed by small RNAs. Cell.

[8] Burgyan J., Havelda Z. (2011). Viral suppressors of RNA silencing. Trends Plant Sci..

[9] Kasschau K.D., Carrington J.C. (1998). A counterdefensive strategy of plant viruses: Suppression of posttranscriptional gene silencing. Cell.

[10] Pumplin N., Voinnet O. (2013). RNA silencing suppression by plant pathogens: Defence, counter-defence and counter-counter-defence. Nat. Rev. Microbiol..

[11] Csorba T., Kontra L., Burgyan J. (2015). viral silencing suppressors: Tools forged to fine-tune host-pathogen coexistence. Virology.

[12] Csorba T., Lozsa R., Hutvagner G., Burgyan J. (2010). Polerovirus protein P0 prevents the assembly of small RNA-containing RISC complexes and leads to degradation of ARGONAUTE1. Plant J..

[13] Chiu M.H., Chen I.H., Baulcombe D.C., Tsai C.H. (2010). The silencing suppressor P25 of *Potato virus X* interacts with Argonaute1 and mediates its degradation through the proteasome pathway. Mol. Plant Pathol..

[14] Merai Z., Kerenyi Z., Molnar A., Barta E., Valoczi A., Bisztray G., Havelda Z., Burgyan J., Silhavy D. (2005). Aureusvirus P14 is an efficient RNA silencing suppressor that binds double-stranded RNAs without size specificity. J. Virol..

[15] Silhavy D., Molnar A., Lucioli A., Szittya G., Hornyik C., Tavazza M., Burgyan J. (2002). A viral protein suppresses RNA silencing and binds silencing-generated, 21- to 25-nucleotide double-stranded RNAs. EMBO J..

[16] Giner A., Lakatos L., Garcia-Chapa M., Lopez-Moya J.J., Burgyan J. (2010). Viral protein inhibits RISC activity by argonaute binding through conserved WG/GW motifs. PLoS Pathog..

[17] Zhuo T., Li Y.Y., Xiang H.Y., Wu Z.Y., Wang X.B., Wang Y., Zhang Y.L., Li D.W., Yu J.L., Han C.G. (2014). Amino acid sequence motifs essential for P0-mediated suppression of RNA silencing in an isolate of potato leafroll virus from Inner Mongolia. Mol. Plant Microbe Interact..

[18] Ye K., Patel D.J. (2005). RNA silencing suppressor p21 of *Beet yellows virus* forms an RNA binding octameric ring structure. Structure.

[19] Li M., Zhang J., Feng M., Wang X., Luo C., Wang Q., Cheng Y. (2018). Characterization of silencing suppressor p24 of *Grapevine leafroll-associated virus 2*. Mol. Plant Pathol..

[20] Sahana N., Kaur H., Jain R.K., Palukaitis P., Canto T., Praveen S. (2014). The asparagine residue in the FRNK box of potyviral helper-component protease is critical for its small RNA binding and subcellular localization. J. Gen. Virol..

[21] Zhou X. (2013). Advances in understanding begomovirus satellites. Annu. Rev. Phytopathol..

[22] Zerbini F.M., Briddon R.W., Idris A., Martin D.P., Moriones E., Navas-Castillo J., Rivera-Bustamante R., Roumagnac P., Varsani A., Ictv Report C. (2017). ICTV Virus Taxonomy Profile: Geminiviridae. J. Gen. Virol..

[23] Harrison B., Robinson D. (1999). Natural genomic and antigenic variation in whitefly-transmitted geminiviruses (Begomoviruses). Annu. Rev. Phytopathol..

[24] Harrison B.D. (1985). Advances in Geminivirus Research. Annu. Rev. Phytopathol..

[25] Hu T., Song Y., Wang Y., Zhou X. (2020). Functional analysis of a novel βV1 gene identified in a geminivirus betasatellite. Sci. China Life Sci..

[26] Mei Y., Yang X., Huang C., Zhang X., Zhou X. (2018). *Tomato leaf curl Yunnan virus*-encoded C4 induces cell division through enhancing stability of Cyclin D 1.1 via impairing NbSKeta -mediated phosphorylation in *Nicotiana benthamiana*. PLoS Pathog..

[27] Yang X., Guo W., Li F., Sunter G., Zhou X. (2019). Geminivirus-associated betasatellites: Exploiting chinks in the antiviral arsenal of Plants. Trends Plant Sci..

[28] Jia Q., Liu N., Xie K., Dai Y., Han S., Zhao X., Qian L., Wang Y., Zhao J., Gorovits R. (2016). CLCuMuB βC1 subverts ubiquitination by interacting with NbSKP1s to enhance geminivirus infection in *Nicotiana benthamiana*. PLoS Pathog..

[29] Ismayil A., Yang M., Haxim Y., Wang Y., Li J., Han L., Wang Y., Zheng X., Wei X., Nagalakshmi U. (2020). Cotton leaf curl Multan virus βC1 protein induces autophagy by disrupting the interaction of autophagy-related protein 3 with glyceraldehyde-3-phosphate dehydrogenases. Plant Cell.

[30] Rosas-Diaz T., Zhang D., Fan P., Wang L., Ding X., Jiang Y., Jimenez-Gongora T., Medina-Puche L., Zhao X., Feng Z. (2018). A virus-targeted plant receptor-like kinase promotes cell-to-cell spread of RNAi. Proc. Natl. Acad. Sci. USA.

[31] Cui X., Li G., Wang D., Hu D., Zhou X. (2005). A Begomovirus DNAβ-encoded protein binds DNA, functions as a suppressor of RNA silencing, and targets the cell nucleus. J. Virol..

[32] Cui X., Zhou X. (2004). AC2 and AC4 proteins of *Tomato yellow leaf curl China virus* and *Tobacco curly shoot virus* mediate suppression of RNA silencing. Chin. Sci. Bull..

[33] Sun M., Jiang K., Li C., Du J., Li M., Ghanem H., Wu G., Qing L. (2020). *Tobacco curly shoot virus* C3 protein enhances viral replication and gene expression in *Nicotiana benthamiana* plants. Virus Res..

[34] Wang B., Yang X., Wang Y., Xie Y., Zhou X. (2018). *Tomato yellow leaf curl virus* V2 interacts with host HDA6 to suppress methylation-mediated transcriptional gene silencing in plants. J. Virol..

[35] Wang Y., Wu Y., Gong Q., Ismayil A., Yuan Y., Lian B., Jia Q., Han M., Deng H., Hong Y. (2019). Geminiviral V2 protein suppresses transcriptional gene silencing through interaction with AGO4. J. Virol..

[36] Rodriguez-Negrete E., Lozano-Duran R., Piedra-Aguilera A., Cruzado L., Bejarano E.R., Castillo A.G. (2013). Geminivirus Rep protein interferes with the plant DNA methylation machinery and suppresses transcriptional gene silencing. New Phytol..

[37] Zhang Z., Chen H., Huang X., Xia R., Zhao Q., Lai J., Teng K., Li Y., Liang L., Du Q. (2011). BSCTV C2 attenuates the degradation of SAMDC1 to suppress DNA methylation-mediated gene silencing in *Arabidopsis*. Plant Cell.

[38] Jing C., Li P., Zhang J., Wang R., Wu G., Li M., Xie L., Qing L. (2019). The *Malvastrum yellow vein virus* C4 protein promotes disease symptom development and enhances virus accumulation in plants. Front. Microbiol..

[39] Castillo-Gonzalez C., Liu X., Huang C., Zhao C., Ma Z., Hu T., Sun F., Zhou Y., Zhou X., Wang X.J. (2015). Geminivirus-encoded TrAP suppressor inhibits the histone methyltransferase SUVH4/KYP to counter host defense. Elife.

[40] Rishishwar R., Dasgupta I. (2019). Suppressors of RNA silencing encoded by geminiviruses and associated DNA satellites. Virusdisease.

[41] Padidam M., Beachy R.N., Fauquet C.M. (1996). The role of AV2 (“precoat”) and coat protein in viral replication and movement in tomato leaf curl geminivirus. Virology.

[42] Poornima Priyadarshini C.G., Ambika M.V., Tippeswamy R., Savithri H.S. (2011). Functional characterization of coat protein and V2 involved in cell to cell movement of *Cotton leaf curl Kokhran virus*-Dabawali. PLoS ONE.

[43] Iqbal Z., Sattar M.N., Kvarnheden A., Mansoor S., Briddon R.W. (2012). Effects of the mutation of selected genes of cotton leaf curl Kokhran virus on infectivity, symptoms and the maintenance of cotton leaf curl Multan betasatellite. Virus Res..

[44] Sharma P., Ikegami M. (2010). *Tomato leaf curl Java virus* V2 protein is a determinant of virulence, hypersensitive response and suppression of posttranscriptional gene silencing. Virology.

[45] Mubin M., Amin I., Amrao L., Briddon R.W., Mansoor S. (2010). The hypersensitive response induced by the V2 protein of a monopartite begomovirus is countered by the C2 protein. Mol. Plant Pathol..

[46] Luna A.P., Morilla G., Voinnet O., Bejarano E.R. (2012). Functional analysis of gene-silencing suppressors from tomato yellow leaf curl disease viruses. Mol. Plant Microbe Interact..

[47] Wang B., Li F., Huang C., Yang X., Qian Y., Xie Y., Zhou X. (2014). V2 of tomato yellow leaf curl virus can suppress methylation-mediated transcriptional gene silencing in plants. J. Gen. Virol..

[48] Zrachya A., Glick E., Levy Y., Arazi T., Citovsky V., Gafni Y. (2007). Suppressor of RNA silencing encoded by *Tomato yellow leaf curl virus*-Israel. Virology.

[49] Sharma P., Ikegami M., Kon T. (2010). Identification of the virulence factors and suppressors of posttranscriptional gene silencing encoded by *Ageratum yellow vein virus*, a monopartite begomovirus. Virus Res..

[50] Amin I., Hussain K., Akbergenov R., Yadav J.S., Qazi J., Mansoor S., Hohn T., Fauquet C.M., Briddon R.W. (2011). Suppressors of RNA silencing encoded by the components of the cotton leaf curl begomovirus-betasatellite complex. Mol. Plant Microbe Interact..

[51] Zhang J., Dong J., Xu Y., Wu J. (2012). V2 protein encoded by *Tomato yellow leaf curl China virus* is an RNA silencing suppressor. Virus Res..

[52] Luna A.P., Rodriguez-Negrete E.A., Morilla G., Wang L., Lozano-Duran R., Castillo A.G., Bejarano E.R. (2017). V2 from a curtovirus is a suppressor of post-transcriptional gene silencing. J. Gen. Virol..

[53] Goodin M.M., Dietzgen R.G., Schichnes D., Ruzin S., Jackson A.O. (2002). pGD vectors: Versatile tools for the expression of green and red fluorescent protein fusions in agroinfiltrated plant leaves. Plant J..

[54] Han Y.H., Xiang H.Y., Wang Q., Li Y.Y., Wu W.Q., Han C.G., Li D.W., Yu J.L. (2010). Ring structure amino acids affect the suppressor activity of melon aphid-borne yellows virus P0 protein. Virology.

[55] Zhao W., Ji Y., Wu S., Ma X., Li S., Sun F., Cheng Z., Zhou Y., Fan Y. (2018). Single amino acid in V2 encoded by TYLCV is responsible for its self-interaction, aggregates and pathogenicity. Sci. Rep..

[56] Liu Q., Guo R., Li M., Feng M., Wang X., Wang Q., Cheng Y. (2016). Critical regions and residues for self-interaction of grapevine leafroll-associated virus 2 protein p24. Virus Res..

[57] Chowda-Reddy R.V., Achenjang F., Felton C., Etarock M.T., Anangfac M.T., Nugent P., Fondong V.N. (2008). Role of a geminivirus AV2 protein putative protein kinase C motif on subcellular localization and pathogenicity. Virus Res..

[58] Saeed F., Sattar M.N., Hameed U., Ilyas M., Haider M.S., Hamza M., Mansoor S., Amin I. (2018). Infectivity of okra enation leaf curl virus and the role of its V2 protein in pathogenicity. Virus Res..

[59] Wartig L., Kheyr-Pour A., Noris E., De Kouchkovsky F., Jouanneau F., Gronenborn B., Jupin I. (1997). Genetic analysis of the monopartite tomato yellow leaf curl geminivirus: Roles of V1, V2, and C2 ORFs in viral pathogenesis. Virology.

[60] Aguilar E., Almendral D., Allende L., Pacheco R., Chung B.N., Canto T., Tenllado F. (2014). The P25 protein of *Potato virus X* is the main pathogenicity determinant responsible for systemic necrosis in PVX-associated synergisms. J. Virol..

[61] Li F., Ding S.W. (2006). Virus counterdefense: Diverse strategies for evading the RNA-silencing immunity. Annu. Rev. Microbiol..

[62] Yang X., Xie Y., Raja P., Li S., Wolf J.N., Shen Q., Bisaro D.M., Zhou X. (2011). Suppression of methylation-mediated transcriptional gene silencing by βC1-SAHH protein interaction during geminivirus-betasatellite infection. PLoS Pathog..

[63] Saeed M., Krczal G., Wassenegger M. (2015). Three gene products of a begomovirus-betasatellite complex restore expression of a transcriptionally silenced green fluorescent protein transgene in *Nicotiana benthamiana*. Virus Genes.

[64] Lakatos L., Csorba T., Pantaleo V., Chapman E.J., Carrington J.C., Liu Y.P., Dolja V.V., Calvino L.F., Lopez-Moya J.J., Burgyan J. (2006). Small RNA binding is a common strategy to suppress RNA silencing by several viral suppressors. EMBO J..

[65] Kontra L., Csorba T. (2016). Distinct effects of p19 RNA silencing suppressor on small RNA mediated pathways in plants. PLoS Pathog..

[66] Merai Z., Kerenyi Z., Kertesz S., Magna M., Lakatos L., Silhavy D. (2006). Double-stranded RNA binding may be a general plant RNA viral strategy to suppress RNA silencing. J. Virol..

[67] Fukunaga R., Doudna J.A. (2009). dsRNA with 5’ overhangs contributes to endogenous and antiviral RNA silencing pathways in plants. EMBO J..

[68] Chen H.Y., Yang J., Lin C., Yuan Y.A. (2008). Structural basis for RNA-silencing suppression by *Tomato aspermy virus* protein 2b. EMBO Rep..

[69] Schwach F., Vaistij F.E., Jones L., Baulcombe D.C. (2005). An RNA-dependent RNA polymerase prevents meristem invasion by potato virus X and is required for the activity but not the production of a systemic silencing signal. Plant Physiol..

[70] Di Serio F., Martinez de Alba A.E., Navarro B., Gisel A., Flores R. (2010). RNA-dependent RNA polymerase 6 delays accumulation and precludes meristem invasion of a viroid that replicates in the nucleus. J. Virol..

[71] Ghoshal B., Sanfacon H. (2015). Symptom recovery in virus-infected plants: Revisiting the role of RNA silencing mechanisms. Virology.

[72] Zhang X.P., Liu D.S., Yan T., Fang X.D., Dong K., Xu J., Wang Y., Yu J.L., Wang X.B. (2017). *Cucumber mosaic virus* coat protein modulates the accumulation of 2b protein and antiviral silencing that causes symptom recovery in planta. PLoS Pathog..

[73] Sunpapao A., Nakai T., Dong F., Mochizuki T., Ohki S.T. (2009). The 2b protein of cucumber mosaic virus is essential for viral infection of the shoot apical meristem and for efficient invasion of leaf primordia in infected tobacco plants. J. Gen. Virol..

[74] Martin-Hernandez A.M., Baulcombe D.C. (2008). *Tobacco rattle virus* 16-kilodalton protein encodes a suppressor of RNA silencing that allows transient viral entry in meristems. J. Virol..

[75] Hormuzdi S.G., Bisaro D.M. (1995). Genetic analysis of beet curly top virus: Examination of the roles of L2 and L3 genes in viral pathogenesis. Virology.

[76] Raja P., Sanville B.C., Buchmann R.C., Bisaro D.M. (2008). Viral genome methylation as an epigenetic defense against geminiviruses. J. Virol..

[77] Raja P., Jackel J.N., Li S., Heard I.M., Bisaro D.M. (2014). *Arabidopsis* double-stranded RNA binding protein DRB3 participates in methylation-mediated defense against geminiviruses. J. Virol..

[78] Coursey T., Regedanz E., Bisaro D.M. (2018). *Arabidopsis* RNA polymerase V mediates enhanced compaction and silencing of geminivirus and transposon chromatin during host recovery from infection. J. Virol..

[79] Cao X., Zhou P., Zhang X., Zhu S., Zhong X., Xiao Q., Ding B., Li Y. (2005). Identification of an RNA silencing suppressor from a plant double-stranded RNA virus. J. Virol..

[80] Li P., Jing C., Ren H., Jia Z., Ghanem H., Wu G., Li M., Qing L. (2020). Analysis of Pathogenicity and Virulence Factors of Ageratum leaf curl Sichuan virus. Front. Plant Sci..

[81] Mason G., Caciagli P., Accotto G.P., Noris E. (2008). Real-time PCR for the quantitation of Tomato yellow leaf curl Sardinia virus in tomato plants and in *Bemisia tabaci*. J. Virol. Methods.

[82] Livak K.J., Schmittgen T.D. (2001). Analysis of relative gene expression data using real-time quantitative PCR and the 2^(−∆∆C(T))^ Method. Methods.

